# Stimulus Features of the Object Relations Technique Affecting the Linguistic Qualities of Individuals’ Narratives

**DOI:** 10.1007/s10936-021-09764-5

**Published:** 2021-01-22

**Authors:** Attà Negri, Martino Ongis

**Affiliations:** 1 Department of Human and Social Sciences, University of Bergamo, Piazzale S. Agostino 2, 24129 Bergamo, Italy; 2 The New School for Social Research, New York, NY USA

**Keywords:** Referential activity, Reorganization function, Stimulus features, Object Relations Technique, Projective test determinants

## Abstract

Previous studies on projective techniques have investigated the effects of variation in stimulus features on individuals’ response behavior. In particular, the influence of chromatic colors and form definition on the images elicited by the stimuli has been tested. Most studies have focused on the Rorschach and TAT and have examined effects in terms of variables such as reality testing and reactions to perceptual details. This is the first study to examine the effects of variation in visual stimuli as represented in features of the Object Relations Technique (ORT) cards on linguistic indicators of connection to emotional experience using measures of the referential process. The ORT was administered to 207 Italian non-clinical participants to explore effects of color, form and content variation on language style. The sample was stratified by age, gender, marital status and education to be representative of the Italian population. The stories told in response to the card images were rated using computerized linguistic measures, including the Weighted Referential Activity Dictionary—Italian version (IWRAD) which indicates the degree to which language is connected to nonverbal experience, and the Weighted Reflection/Reorganization List—Italian version (IWRRL) which detects a linguistic style of personal re-elaboration of emotional experience. The results provide support for the color-affect and form-reality testing hypotheses. Cards with better form definition, including color definition, and with fewer silhouettes of people elicited responses that were higher in IWRAD and lower in IWRRL, and also higher in the degree to which the two measures varied together. Implications of the results for use of ORT in clinical assessment and intervention are discussed.

## Introduction

Projective techniques have been understood in a variety of different ways since they were first conceived. In early applications, at a time when the Freudian model was the dominant theoretical framework, projectives were interpreted primarily as instruments that were able to circumvent defenses and conscious control of the patient and to provide access to contents that were not otherwise accessible (Murray 1938; Frank 1939). The ambiguity of the stimuli in these tests was understood to stimulate the projection of the internal world, avoiding the perception of reality as much as possible. For this reason they were named *projective* techniques or tests, and were intended to offer a more objective evaluation than the therapist's impressions and to enable more reliable and objective diagnoses or case formulations to inform treatment choices.

These techniques fell out of favor with the increasing popularity of ‘evidence based’ psychotherapy approaches, which emphasized directly observable aspects of personality. Projective methods were no longer considered reliable or valid and several studies pointed out the theoretical and subjective biases in the coding and interpretation of these systems as well as their poor predictive validity with respect to real-life behavior (e.g. Baughman 1958, 1967; Wood et al. 2003).

More recently, projective techniques have been finding new uses in clinical practice and a renewed scientific status. New approaches to psychological intervention have shifted the focus of the investigation from individual’s internal psychic structures to meaning making processes and to human subjectivity emerging in the relationship with patients, even by means of the administration of psychological tests (for example Fischer 1994; Finn 2007). Many relational approaches to psychotherapy (e.g. Mitchell 1988; Safran and Muran 2000; Bromberg 2011) call for the reactivation of typical functioning modalities of the patient as a central and necessary component of the change process. Re-experiencing the emotional, cognitive and interpersonal processes that cause suffering to a person in the context of the therapeutic relationship help to put these painful experiences into words, so as to better understand them, and open paths of change.

From this point of view projective techniques may potentially operate as ways of activating emotional functioning in the here and now of the therapeutic relationship. Supporting this perspective, some authors (Kubiszyn et al. 2000; Meyer et al. 2001) define projective tests as performance based personality tests; that is, as specific perceptual problem solving situations in which individuals have to decide how to respond, activating processes similar to those of everyday functioning. As Kubiszyn et al. (2000) state: “More often than not, the information obtained from these tasks reflects the patient's perceptions, classifications, and cognitive-emotional templates or internal representations, rather than projections per se” (p. 120). Stated a bit differently, the ambiguity and vagueness of the stimuli are viewed as activating perceptual problem-solving processes in the moment rather than as uncovering unconscious contents that have previously been formed.

### Affectivity and Reality Testing in the Projective Techniques

The empirical investigation we present in this paper is based on two related hypotheses, originally proposed by Rorschach (1921/1942) and subsequently supported by several authors. The color-affect hypothesis states that there is a connection between stimulus chromatic features, color based responses and affective regulation capability; the form-reality testing hypothesis claims that an association exists between the degree of stimulus contour definition, accurate recognition of specific forms, and reality testing.

#### Color-Affect Hypothesis

Many projective tests such as the Rorschach Inkblot Method (RIM) are comprised of achromatic (black, white, grey) and chromatic cards. Rorschach (1921/1942) hypothesized that (a) the chromatic colors in inkblots elicited “emotional excitability and actual excitement” (p. 98) and (b) the responses including chromatic color were representative of “the total affective responsiveness” (p. 192). The first part of the hypothesis concerns the degree to which color activates more emotional responses; the second part concerns the degree to which the colored images in the response are indicators of the individual's emotional functioning. In particular, the degree to which color was associated with a specific form was considered a measure of the ability to regulate and control affects and emotions; the more focus on colored percepts without a defined shape, the greater the impulsivity and emotional lability of the individual.

Studies investigating the first part of this hypothesis have shown mixed results. There is, however, some evidence that achromatic cards are more likely to elicit emotionally inhibited responses and depressive themes whereas chromatic cards are more likely to arouse emotional involvement (Siipola 1950; Exner 1959; Exner and Depauw 1962; Drechsler 1960; Crumpton and Groot 1966; Ray 1975; Silva and Ferreira 2014). For example, chromatic cards have been found to produce a higher number of responses, a longer reaction time, and to be evaluated as more pleasant or unpleasant than achromatic ones. They also tend to elicit more emotionally charged contents and have been found to be more likely to activate the left orbitofrontal cortex—widely involved in emotion regulation—than the achromatic cards (Ishibashi et al. 2016).

The second part of the hypothesis has also been investigated in several studies testing the relationship of the responses including color with impulsivity and affective instability. Emotional disorganization is greater when the chromatic color is central in the response but is not associated with a specific form. For example, Malone et al. (2013) found that images whose primary determinant is color associated only secondarily to a form (CF) correlated with broad-ranging psychopathology, suicidality, emotional dysregulation, self-harm, aggression, and neuroticism; colored images without any form (C) positively correlated with negative affect, paranoia, social isolation, lower extraversion and openness. Mihura et al. (2003) found that the degree of connection between color and form in the responses was negatively associated with self-reported physical aggression potential, suicidal ideation with impulsivity, and borderline features. The meta-analyses conducted by Mihura et al. (2013) also reported similar findings. For example, the proportion of colored images in the responses (affective ratio) was significantly related to affective situations and conditions (e.g. eating rate of obese patients’; diagnosis of borderline PD; identification of sex offenders and psychopaths). The extent to which the color was associated to a specific form (Form-Color Ratio) was inversely associated to indicators of emotional impulsivity or reactivity (e.g. acute stress reactions, PTSD and suicide attempts) and was also lower in substantiated child-abusing fathers, male violent offenders, and patients hospitalized for psychosis compared to non-patients).

#### Form-Reality Testing Hypothesis

The indefiniteness of contours and the lack of sharpness of the images used in most projective tests were originally conceived as expedients to stimulate projection of unconscious needs, feelings and functions. More recently, as we have mentioned, these tests are now considered primarily as perceptual problem solving tasks in which the individual, starting from an ambiguous stimulus, has to choose or create defined images to describe, or around which to construct a narrative. Rorschach (1921/1942) considered the perception of form an essential component of the RIM, originally naming his ten cards “the form interpretation test.” In his view: (a) the greater the degree of sharpness of the visual stimuli administered, the easier it would be to visualize specific and accurate forms, and (b) the visualization of images, when they are sharp, accurate and correspond to the stimuli contours, is an index of intelligence and reality testing. In his terms, the correspondence between the forms imagined and the stimuli provided is a measure of “‘fonction du réel, the practiced, conscious logic of adaptability” (p. 80).

Some empirical studies (Klein and Arnheim 1953; Baughman 1954, 1958; Belden and Baughman 1954) confirmed the influence of the degree of sharpness on the form’s visualization: the more definite, structured, well-defined and familiar the stimulus, the more likely the individual is to produce answers with detailed and accurate forms corresponding to the characteristics of the stimulus.

To examine the effects of correspondence between critical features of stimuli and images perceived as indicators of reality testing, several empirical studies have compared responses by patients with various thinking disorders with those of non-clinical individuals. For example, Bodoin and Pikunas (1983) found a significantly lower percentage of responses to colored images fitting well to the stimulus features (FC+%), in all groups of psychiatric patients considered (borderline, manic-depressive, schizo-affective, and schizophrenic) compared to the non-patient group. Bartell and Soltano (1995) found that children with ADHD described images with a poor form quality (X−%) when compared to norms. Hilsenroth et al. (2007) detected significantly more disordered thinking as measured by the Perceptual Thinking Index in patients suffering from psychotic, conduct and personality disorders when compared with a non-clinical group. Based on these and others similar results the main RIM coding systems (Exner 2003; Meyer et al. 2011) consider the indices relating to the form perception and its quality (e.g. Perceptual Thinking Index, Ego Impairment Index, F+%, X+%) as measures of logical thought, adjustment capability and reality testing.

### Linguistic Qualities as Measures of Psychological Functioning

In this study we go beyond the idea of reality testing as such and take the point of view that the task of telling a story or describing images in response to specific visual stimuli is to a large extent a perceptual problem solving task that highlights an individual’s perceptual, cognitive, emotional and interpersonal style through which they build and maintain relationships in their everyday life. These processes can also contribute to the development of the therapeutic relationship and to the activation of core emotional and interpersonal schemas. Through the narratives or descriptions that are elicited, useful information may be obtained for diagnosis and also for understanding these processes, and as a basis for change.

The ORT, used in this study, was designed to address such an application of projective testing, as will be discussed below. The view of response to perceptual stimuli as indicators of emotional and interpersonal functioning is also compatible with the Multiple Code Theory (MCT; Bucci 1997; Bucci et al. 2016), an innovative theory of mental functioning that hypothesizes a link between language qualities and properties of an individual’s processing of information, including emotional information. As described in detail by Bucci (2021a), MCT proposes the existence of three systems through which people process information—the subsymbolic, non-verbal symbolic and verbal symbolic systems. Connections among these systems take place via the *referential process* whereby sensory, visceral and emotional components of experience (at the subsymbolic level) are represented as images (in all modalities) and then connected to language. The subsymbolic system processes information in global and analogical ways in all sensory modalities and in visceral and kinesthetic experience. The information processed through this system cannot be segmented into discrete elements but is coded along continuous dimensions. The non-verbal symbolic system operates with discrete images or representations that take shape from the continuous flow of subsymbolic experience. Finally, in the verbal symbolic system these images and representations can be represented in words, even if only partially.

Emotional organization depends on integration of these systems, connections of subsymbolic bodily experience to experiences involving people and events as these occur throughout life. Adequate emotional and interpersonal functioning depends on such integration; in emotional disorders these connections may be disrupted or dissociated (see Bucci 2021a).

The referential process refers to the connection of the systems; psychotherapy normally reactivates and strengthens such process through the following three functions that often occur sequentially within a session: (a) *arousal* of subsymbolic aspects of an emotion schema; (b) *symbolization* in which the patient can express an activated emotion schema verbally in the form of a narrative of an event, or a memory, dream or fantasy; and (c) *reflection and reorganization* of the narrative that has been told; this leads to finding and creating new meaningful connections among the three systems (Bucci 1997, 2021a; Bucci et al. 2016).

Over the last 20 years, several linguistic measures have been developed and validated in different languages to visualize, monitor, and measure these functions of the referential process. The Symbolizing function is represented by the *Referential Activity* (RA) measure (WRAD) and measures derived from that. The Reflection and Reorganization function (RR) was first measured by the unweighted *Reflection* dictionary (REF); more recently a second measure of this function, the *Weighted Reflection/Reorganization List* (WRRL), has been developed:

(i) RA can be defined as the degree to which the speaker or writer is able to translate his emotional, visceral and relational experience into words, so as to evoke corresponding experiences in the listener or reader. As discussed by Bucci (2021b) RA was initially measured using scales of *Concreteness, Specificity, Clarity, and Imagery,* rated by judges, following instructions given in a manual (Bucci and Kabasakalian-McKay 1992).^1^ To allow reliable measurement of RA in large samples and long-term treatments, computerized measures were developed. The measure currently in use is the Weighted Referential Activity Dictionary (WRAD; see Maskit 2021). An Italian version of this measure, the IWRAD has been developed by Mariani et al. (2013).
(ii) RR can be defined as the degree to which the speaker is trying to recognize and understand the emotional significance of an event or set of events in their own or someone else’s life, or in a dream or fantasy (see Bucci 2021a). The first computer measure of the RR function was provided by the unweighted Reflection dictionary (REF), a list of words developed using standard procedures of computer assisted content analysis. Items in this dictionary include words that refer to cognitive or logical functions and expressions, logical problems, and mental and communicative functions (see Maskit 2021). The Italian version of the REF, the IREF, was initially developed by Mariani (2009); also see Mariani et al. (2013). While there is evidence that the REF dictionary is associated with the Reflecting and Reorganizing function, as presented by Maskit, the coverage is generally in the range of 8–10%, considerably lower than the WRAD coverage. Also, the REF is often interpreted as assessing abstract and logical functioning, which may also involve attempts to avoid emotional experience, rather than to confront and attempt to regulate such experience. To address problems associated with the REF, a second measure of this function, the Weighted Reflecting/Reorganizing List (WRRL), was developed using a modified version of the procedure used to develop the WRAD (see Zhou et al. 2021). An Italian version of this measure, the IWRRL has been developed by Negri et al. (2018).

Whereas the REF assesses linguistic contents associated with abstract thought, the WRRL and IWRRL, like the WRAD and IWRAD, primarily concerns linguistic style rather than contents, *how* people talk rather than *what they talk about.* IWRAD allow detecting in a therapy session or in a speech the symbolization phase, IWRRL the reflection and reorganization one (See Bucci 2021a). Other complementary measures of the referential process have also been developed that refer primarily to contents rather than style of speech. Like the REF, they detect the proportion of words present in a speech sample related to specific themes connected in some way to the RP. These may include words referring to affects, and sensory-somatic issues, and also include measures of disfluency (see Maskit 2021). Some of these alongside the IWRAD and the IWRRL will be applied in this study to narratives elicited by the Object Relations Technique, a narrative projective test chosen for its unique and specific stimulus features.

### Object Relations Technique

Described by Grotstein as “an innovative and unique descendent of the heritage of projective testing on one hand and of projective aspects of psychoanalytic clinical technique on the other” (1993, p. 1), the Object Relations Techniques (ORT; Phillipson 1955; Shaw 2002; Knafo 2010) was originated by Herbert Phillipson at the Tavistock Institute to investigate people’s interpersonal inner worlds from the perspective of object relations theory. Phillipson conceived ORT as a test integrating the main characteristics of the two most common projective tests, Rorschach Inkblot Method (RIM) and Thematic Apperception Test (TAT). From RIM he retained the perceptual features that have been shown to detect specific aspects of psychological functioning such as the variation of form vagueness, achromatic and chromatic colors and chiaroscuro tonalities involving contrasts of light and shading; from TAT he kept the innovation of showing human figures in various contexts to facilitate the projection of aspects of the self and relationships, as well as the instruction of telling a story and not just describing the images elicited by the cards. This combination resulted in a very rich test. On one hand, ORT goes beyond the abstractness and unfamiliarity of the RIM figures while keeping its perceptual complexity; on the other, it transcends the TAT’s use of only achromatic drawings while retaining its innovation of human figures in the stimuli. Finally, unlike the TAT that presents very disparate interpersonal settings among which the clinician chooses the most useful ones for working with the specific patient, ORT comprises a fixed number of cards that explore in a systematic way several major interpersonal settings and that are administered in a specified order.

The ORT consists of twelve cards with human silhouettes and objects, and a final blank card. Following the instructions provided by Lis et al. (2002) the participants are asked to tell a story for each card without content restrictions or time limits. As shown in Table 1 the cards can be divided into three group—A, B and C—which correspond to three levels of variation on two dimensions: (1) the progressive increase of definitions and reality contents of the images (from vague images to drawings that are well defined with clear stimuli contours, commonly-recognizable objects, and setting details); and (2) the achromatic-chromatic colors continuum (from mainly gray, to black and white, and to multicolored cards). An additional dimension is transversal to the previous ones: within each group we find a 1-person, a 2-person, a 3-person and a group card. The thirteenth card, the last one, is blank and can be placed at the zero level of the three dimensions.

**Table 1:** Stimulus features and psychological aspects elicited by the ORT plates

	Cards groups (stimulus type)		
	A	B	C
Stimulus features	Vague contours	Defined contours	Defined contours
	Achromatic colors	Achromatic colors	Chromatic colors
	Shades	No shades	Shades
	Bare setting	Setting details	Setting details
Interpersonal settings	1-person (1)	1-person (6)	1-person (12)
	2-person (2)	2-person (9)	2-person (11)
	3-person (8)	3-person (4)	3-person (3)
	Group (5)	Group (10)	Group (7)
Aspects elicited	Infancy	Latency	Maturity
	Anxiety, uncertainty, dependence	Loss, deprivation, hostility	Various and complex affects

The order of presentation is shown in parentheses. The table does not include the 13th card, which is blank

## Aims and Hypotheses

In this study we sought to explore the psychological processes activated by the different properties of cards in the ORT by measuring the variation in linguistic style of narratives elicited by them. In particular, we sought to investigate the effect of the chromatic colors and the progressive increase in the reality cues of contour definition and setting details on the language of the participant’s responses measured using the computerized versions of RA (IWRAD) and RR (IWRRL) and other dictionaries.

Considering that the chromatic color cards should prompt greater emotional involvement—as stated by the color-affect hypothesis—and that clearer stimulus contours should elicit more specific, clear, and better defined responses—as claimed by form-reality testing hypothesis—we hypothesized that the IWRAD level of the narratives elicited by the cards would progressively increase from the blank card to Group A, B, and C of the ORT cards. In contrast, we expect that personal reflection and reorganization of the emotional experience as measured by IWRRL will be highest in the blank card, where the individual has no perceptual constraints and must turn inward in his responses and will be lower as the perceptual constraints increase*.*

More in detail, based on the hypotheses of Phillipson (1955) and Lis et al. (2002), we have the following expectations:

- The blank card will elicit narratives that are relatively low in IWRAD and relatively high in IWRRL. The absence of perceptual constraints should favor less connection to specific experiences and more reflecting.
- The narratives elicited by the cards in Group A will also be relatively low in IWRAD and relatively high in IWRRL. The vague contours and bare setting with limited perceptual details provide limited reality structure. Furthermore, the cards are achromatic, thus less likely to be evocative of emotion; the presence of shadings would further limit reality constraints, thus favoring an inward based response.
- The cards of Group B will elicit narratives with medium levels of IWRRL and IWRAD; on the one hand the more well-defined contours, with no shading, and more reality details define the setting more clearly; on the other hand the cards are achromatic and thus less activating of emotion.
- The cards of Group C, with defined contours and reality details will elicit narratives with higher levels of IWRAD and lower levels of IWRRL. In addition, the chromatic colors should elicit more emotional activation linked to specific contexts. (These factors are expected to override any effect of shadings).

The ORT cards also vary systematically in the number of human silhouettes outlined; the effects of variation in this property are not predicted and will be explored. The effects of the cards on other linguistic measures will also be explored.

## Method

### Participants

Participants (N = 207) from a non-clinical Italian sample were stratified by gender, age, marital status and education proportionally to the real Italian population as sampled by the Italian National Institute of Statistics (Istituto Nazionale di Statistica 2016): gender (women = 106; man = 101), age (*M* = 40.9 years; *SD* = 11.0 years), marital status (71 singles; 121 cohabitants; 15 divorced or widowed), years of education (*M* = 11.7; *SD* = 3.6, range = 5–22). We also measured Social Status according to the Hollingshead classification (1975; *M* = 29.5; *SD* = 15.8, range = 3–76). The participants were recruited via community outreach (e.g., referrals, snowball sampling) in the northern Italy area.

### Measures

#### Symptom Checklist-90-Revised (SCL-90-R; Derogatis 1994)

The SCL-90-R is a 90-item self-report symptom inventory that assesses psychological distress in terms of nine primary symptom dimensions (Somatization, Obsessive–Compulsive, Interpersonal Sensitivity, Depression, Anxiety, Hostility, Phobic Anxiety, Paranoid Ideation, and Psychoticism) and three summary scores termed global scores (Global Severity Index, the Positive Symptom Distress Index, and the Positive Symptom Total).

#### Computerized Measures of Referential Process (Bucci 2002; Bucci and Maskit 2006; Mariani et al. 2013; Maskit 2014; Maskit 2021)

The Italian Discourse Attributes Analysis Program (IDAAP) is a version of the DAAP program (Maskit 2021). The program compares each word of a text with a weighted or unweighted dictionary and produces the smooth dictionary measure, whose graph is a visually smooth curve that describes the extent to which the speaker is involved in the psycholinguistic process represented by the particular dictionary. For two different such dictionaries, DAAP uses these smooth dictionary measures to produce their covariation, the extent to which the two measures rise and fall together. In our study, we used the following IDAAP dictionaries as indicators of the referential process:

- *Italian Weighted Referential Activity Dictionary (IWRAD)*
  IWRAD is a dictionary of Italian words that models judges’ scoring of RA by assigning a weight to each word that corresponds to relatively higher, or lower RA as scored by judges. IWRAD scores range from 0 (lowest) to 1 (highest RA).
- *Italian Weighted Reflection and Reorganization List (IWRRL)*
  IWRRL is a dictionary of Italian words that models judges’ scoring of RR by assigning a weight to each word that corresponds to relatively higher, or lower RR as scored by judges. IWRRL scores range from 0 (lowest) to 1 (highest RR).
- *Italian Reflection Dictionary (IRefD)*
  IRefD consists of Italian words referring to cognitive or logical functions, and to communication processes that imply the use of cognitive functions.
- *Italian Affect Dictionary (IAffD)*
  IAffD consists of three sub-dictionaries related to affect: Positive Affect (IPAffD), Negative Affect (INAffD), Neutral Affect without a specific valence (IZAffD).

The IDAAP can also calculate the co-variations between the linguistic measures of interest. In our study we take into account the IWRAD_IWRRL co-variation that is a measure of the extent to which IWRAD and IWRRL are simultaneously high or low during the storytelling. This co-variation is of particular importance to detect the activation of the referential process during the speech; it can be viewed as an indicator of the extent to which the speaker is emotionally engaged in describing an image or telling a story (high IWRAD) and also finding a personal meaning of this story (high IWRRL), rather than reflecting on it in an abstract manner.

All linguistic measures produced by IDAAP are averages rather than sums. They are thus not affected by differences in length of narratives told to the ORT that as per instructions they have no time and content limits.

### Procedures

Participants were asked to participate individually in a study on validity of a projective test, the ORT. The test was administered by four trained graduate students in a suitable room on the University of Bergamo campus. Before administration a written consent to participate and be audio-recorded during the study was provided. At the end, a debriefing on the study was given to participants.

Verbatim transcriptions were made of all narratives told in response to the ORT cards in order to estimate the linguistic qualities of each story through the IDAAP application. Because of possible validity issues (Lis et al. 2002) we excluded from the analyses ORT protocols with fewer than 500 words. Since the study called for a non-clinical sample, we also excluded participants who scored higher than the clinical cutoff value on the Global Severity Index of the SCL-90-R.

### Data Analysis

Two stimulus characteristics of the ORT were considered as independent variables: (a) chromatic variation along with the degree of definition, and (b) number of people depicted on the cards (see Table 1). The presence of significant effects on dependent variables—the linguistic measures of referential process—was verified through a series of 4 × 5 (blank, A, B, and C cards groups × 0-person, 1-person, 2-person, 3-person, group cards) repeated measures ANOVAs for each linguistic variable considered.

Gender and marital status have not been included in the ANOVA models since no significant differences were found in all dependent variables between women and men, and between the various marital status categories.

Age, years of education, Social Status Index (Hollingshead 1975) and Global Severity Index (SCL-90-R) were evaluated as covariates, but as they did not show any significant effect they were removed from the final set of analyses.

## Results

We found significant effects of the ORT stimulus features we considered as independent variables—chromatic variation along with the degree of definition, and number of people—on the referential process linguistic qualities of narratives elicited by cards (see Table 2).

**Table 2:** The stimulus features effects on linguistic measures estimated by repeated measures ANOVAs models

	Effects	Λ	*F*	*df*	*p*	η^2^_p_
IWRAD	Cards groups	.472	75.346	3, 202	.000	.528
	N-person	.545	41.926	4, 201	.000	.455
	Interaction	.313	35.278	12, 193	.000	.687
IWRRL	Cards groups	.923	5.638	3, 202	.001	.077
	N-person	.880	6.866	4, 201	.000	.120
	Interaction	.644	8.880	12, 193	.000	.356
IWRAD_IWRRL	Cards groups	.527	60.416	3, 202	.000	.473
	N-person	.572	37.621	4, 201	.000	.428
	Interaction	.420	22.166	12, 193	.000	.580
IRefD	Cards groups	.759	21.425	3, 202	.000	.241
	N-person	.834	10.010	4, 201	.000	.166
	Interaction	.516	15.064	12, 193	.000	.484
IPAffD	Cards groups	.774	19.612	3, 202	.000	.226
	N-person	.618	31.024	4, 201	.000	.382
	Interaction	.489	16.830	12, 193	.000	.511
INAffD	Cards groups	.937	4.507	3, 202	.004	.063
	N-person	.874	7.267	4, 201	.000	.126
	Interaction	.697	6.996	12, 193	.000	.303
IZAffD	Cards groups	.963	2.565	3, 202	.054	.037
	N-person	.949	2.698	4, 201	.032	.051
	Interaction	.852	2.789	12, 193	.002	.148

*IWRAD* Italian Weighted Referential Activity Dictionary, *IWRRL* Italian Weighted Reflection and Reorganization List, *IWRAD_IWRRL* co-variation between IWRAD and IWRRL, *IRefD* Italian Reflection Dictionary, *IPAffD* Italian Positive Affect Dictionary, *INAffD* Italian Negative Affect Dictionary, *IZAffD* Italian Neutral Affect Dictionary

For the first independent variable, planned contrasts revealed that all comparisons between Group A, B, C of cards were significant for the IWRAD and IWRRL variables, and for the co-variation of IWRAD and IWRRL (see Table 3). The average IWRAD values of narratives increased progressively from the blank card to the Group A, *F*(1) = 18.809, *p* = .000, η^2^_p_ = .084, from Group A to Group B, *F*(1) = 77.905, *p* = .000, η^2^_p_ = .276, and from Group B to Group C, *F*(1) = 12.380, *p* = .000, η^2^_p_ = .057. The average IWRRL scores for the blank card were significantly higher than for each of the other card groups, Group A, *F*(1) = 9.178, *p* = .003, η^2^_p_ = .043; Group B, *F*(1) = 11.103, *p* = .002, η^2^_p_ = .046; Group C, *F*(1) = 8.339, *p* = .004, η^2^_p_ = .039; differences among the other groups were not notable. The co-variation IWRAD_IWRRL values had the same trend of the IWRAD scores: They increased progressively from the blank card to the Group A, *F*(1) = 34.178, *p* = .000, η^2^_p_ = .143, from Group A to Group B, *F*(1) = 52.718, *p* = .000, η^2^_p_ = .205, and from Group B to Group C, *F*(1) = 7.016, *p* = .009, η^2^_p_ = .033.

**Table 3:** Descriptive statistics of the linguistic measures by cards groups and interpersonal settings

	Cards groups (stimulus type)—*M (SD)*			
	Blank	A	B	C
IWRAD	.5021 (.0108)	.5053 (.0057)	.5088 (.0058)	**.5102** (.0054)
IWRRL	**.5484** (.0090)	.5465 (.0046)	.5459 (.0041)	.5465 (.0043)
IWRAD_IWRRL	.0756 (.7147)	.3724 (.4219)	.5821 (.3373)	**.6479** (.3000)
IRefD	.0289 (.0228)	**.0352** (.0167)	.0266 (.0139)	.0281 (.0144)
IPAffD	**.0330** (.0415)	.0165 (.0117)	.0182 (.0117)	.0146 (.0108)
INAffD	.0064 (.0102)	.0088 (.0083)	**.0096** (.0082)	.0092 (.0074)
IZAffD	**.0054** (.0115)	.0041 (.0049)	.0041 (.0048)	.0035 (.0041)

	Interpersonal settings—*M (SD)*				
	Blank	1-person	2-person	3-person	Group
IWRAD	.5021 (.0108)	**.5106** (.0064)	.5086 (.0059)	.5070 (.0060)	.5062 (.0058)
IWRRL	**.5484** (.0090)	.5460 (.0047)	.5460 (.0048)	.5460 (.0048)	.5473 (.0052)
IWRAD_IWRRL	.0756 (.7147)	**.6507** (.3154)	.5752 (.3484)	.4586 (.4244)	.4544 (.4162)
IRefD	.0289 (.0228)	.0273 (.0147)	.0276 (.0147)	**.0336** (.0168)	.0313 (.0167)
IPAffD	**.0330** (.0415)	.0141 (.0127)	.0242 (.0169)	.0148 (.0101)	.0127 (.0107)
INAffD	.0064 (.0102)	.0101 (.0095)	.0077 (.0077)	.0088 (.0087)	**.0103** (.0091)
IZAffD	**.0054** (.0115)	.0034 (.0047)	.0043 (.0055)	.0036 (.0045)	.0042 (.0057)

*IWRAD* Italian Weighted Referential Activity Dictionary, *IWRRL* Italian Weighted Reflection and Reorganization List, *IWRAD_IWRRL* co-variation between IWRAD and IWRRL, *IRefD* Italian Reflection Dictionary, *IPAffD* Italian Positive Affects Dictionary, *INAffD* Italian Negative Affects Dictionary, *IZAffD* Italian Neutral Affects Dictionary

In bold the significantly higher values in the comparisons

In sum, as the degree of definition and the chromatic features of the cards increased, the narratives told in response were more vivid, specific, clear and concrete. The tendency to reflect in a non-abstract way on the stories did not increase systematically with these variations in features of the cards; however the association of such reflection with the more vivid narratives did show a significant and systematic increase, which had not been predicted and which will be discussed further. The blank card appeared to elicit personal reflection but with less immediate emotional involvement.

The level of IWRAD and IWRRL of narratives, and the co-variation values between IWRAD and IWRRL changed significantly as the number of human silhouettes sketched on the cards varied (see Tables 2, 3). For the IWRAD values all comparisons but one are significant in the predicted direction. Narratives developed from cards depicting one individual have significantly higher IWRAD scores than the ones developed from cards with two individuals, *F*(1) = 20.257, *p* = .000, η^2^_p_ = .090; in turn, these latter have significantly higher IWRAD scores than the narratives developed from cards with three individuals, *F*(1) = 12.901, *p* = .000, η^2^_p_ = .059; the difference between IWRAD levels elicited by cards with three individuals and group cards is marginally significant, *F*(1) = 3.400, *p* = .067, η^2^_p_ = .016, while the difference between the group cards and the blank one is significant, *F*(1) = 29.187, *p* = .000, η^2^_p_ = .125. The average IWRRL scores decreased significantly from the blank card to the 1-person cards, *F*(1) = 13.175, *p* = .000, η^2^_p_ = .061, while between 1-person, 2-person, 3-person cards no differences were detected. The group cards IWRRL average was significantly higher than the 1-person cards, *F*(1) = 9.295, *p* = .003, η^2^_p_ = .044, 2-person cards, *F*(1) = 9.984, *p* = .002, η^2^_p_ = .047, and 3-person cards, *F*(1) = 12.106, *p* = .001, η^2^_p_ = .056; and lower than the blank card; here the difference approached but did not reach significance, *F*(1) = 2.940, *p* = .088, η^2^_p_ = .014. The IWRAD_IWRRL co-variation values progressively decreased from 1-person cards to 2-person cards, *F*(1) = 9.141, *p* = .003, η^2^_p_ = .043, from 2-person cards to 3-person cards, *F*(1) = 12.819, *p* = .000, η^2^_p_ = .059, from 3-person cards to group cards even if non significantly, and from group cards to blank card, *F*(1) = 56.511, *p* = .000, η^2^_p_ = .217. This covariation was near zero for the blank card; this result will be discussed further.

In sum, as the number of individuals depicted on the cards increased, the average IWRAD level of the narratives as well as the co-variation IWRAD_IWRRL decreased, and both IWRAD and the IWRAD_IWRRL co-variation had the lowest average values in the blank card. The IWRRL showed higher scores in the blank card in comparison with all other groups of cards; the difference was not statistically significant for the group cards.

A significant interaction effect between number of human silhouettes and degree of figure definition was also found for IWRAD, IWRRL, and IWRAD_IWRRL (see Table 2): As for IWRAD and IWRAD_IWRRL the progressive increase of the scores from Group A to Group C was more marked in 1-person cards and progressively less in the other interpersonal settings; in contrast, the progressive decrease of the IWRRL scores from Group A to Group C was more marked in 1-person cards and progressively less in the other interpersonal settings.

Although we did not formulate specific hypotheses concerning the other complementary linguistic measures of interest, we found significant effects that are worth reporting (see Tables 2, 3). The number of words referring to abstract thought and reflection (IRefD) shows the highest value in Group A and in the 3-person cards; the words relating to affects with positive valence (IPAffD) and neutral valence (IZAffD) are present in the highest degree in the blank card with respect to both the other interpersonal settings and the groups of cards A, B and C. The words related to the negative affects (INAffD) are present with greater frequency during the administration of the cards of Group B and the group situation cards.

## Discussion

This is the first study to examine the effects of variation in visual stimuli as represented in features of the ORT cards on linguistic indicators of connection to emotional experience using measures of the referential process. The hypothesis that particular stimulus features of the ORT cards affect the linguistic qualities of the narratives inspired by the cards has been supported by the results. In particular, the variation from achromatic to chromatic cards, the increase in details of reality, and the greater definition of the figure contours elicit narratives with higher referential activity, measured by IWRAD. We suggest that such stimulus features may activate specific sensory, emotional and cognitive processes as represented in more vivid and immediate instantiations of emotion schemas (Bucci 1997; Bucci et al. 2016). We suggest that the color-affect and form-reality testing hypotheses that have been widely proposed in the literature also obtain new support through the linguistic measures applied here.

### Support for the Color-Affect Hypothesis

The higher level of RA, as measured by IWRAD, in the narratives prompted by the chromatic color cards (Group C), shows the effects of color in activating sensory and affective experience as expressed in language style. This finding supports Rorschach’s idea (1921/1942) that chromatic colors have specific effects on the emotional inner world, as well as Schachtel’s claim (1966) that chromatic colors are directly related to affective experience, and that the images are not only recognized but felt as well.

In our view, several additional results provide indirect support for the color-affect hypothesis. First, the blank card is associated with higher levels of the neutral and positive affect dictionaries, while the Group B cards, showing achromatic colors and defined contours, stimulate narratives with the highest negative affect levels. In contrast, responses to the chromatic cards (Group C) did not show high levels of the affect dictionaries, but were highest in IWRAD and the IWRAD_IWRRL covariation, as noted above. These results might appear to contradict the color-affect hypothesis. As several studies have shown, however, using abstract words to name affects, rather than describing the emotional experience in a vivid, specific, and concrete way is often used as a defensive and distancing action (Lieberman et al. 2007; Tabibnia et al. 2008; Izard et al. 2008; Bucci et al. 2016). It follows that high values of the affect dictionaries (IPAffD, INAffD, IZAffD) may suggest attempts to distance or control connection with emotional experience. We speculate that the black and white outlines in the Group B cards may elicit issues of deprivation, loss, hostility and death, while the blank card, with its challenging task of imagining a story without perceptual prompts, may activate functions of control and coping with one’s ideals and future self (Phillipson 1955). We note that this speculation is also supported by the relatively high level of the IWRRL, while WRAD is relatively low in responses to the blank card, indicating processes of internal organization of emotional experience, rather than direct immersion in such experience.

We also note that the level of the reflection dictionary (IRefD) is greater in responses to the vaguely contoured achromatic cards of Group A than any of the other conditions, suggesting stronger defensive operations towards emotions in these cards. The dependency, anxiety and uncertainty issues prompted by this group of cards probably stimulate a higher level of reflection and defense. Overall the data provide confirmation for the effects of chromatic colors in eliciting greater emotional involvement as expressed both in vivid and detailed narratives of experiences combined with some organization of their emotional meanings.

### Support for the Form-Reality Hypothesis

The gradual and significant increase of IWRAD in the narratives, starting from the blank card up to the Group C cards, also provides support for the form-reality hypothesis. The degree of details, shapes, outlines and settings is progressive and corresponds to the increase registered in the IWRAD measure.

According to many current views, projective techniques are primarily a task of perceptual problem solving linked to the recognition of forms (Kubiszyn et al. 2000; Meyer et al. 2001). In the main scoring systems of projective techniques the image-stimulus correspondence is viewed as a measure of adaptation and reality testing. The significant positive correlation between form definition of the stimuli and the IWRAD levels of the narratives that were elicited suggests that these linguistic qualities can also be considered as indirect indicators of such adaptive capacities.

The findings of this study support our claim that greater definition of contours and more details in the drawings are associated with higher levels of concreteness, specificity, clarity and vividness of the narratives elicited by stimuli. We suggest that the degree of correspondence between the stimuli administered and images or narrations given in response may be seen as a measure of reality testing and adaptation to the environment, along with the recognition of forms themselves.

### Suggestions for Clinical and Therapeutic Assessment

If projective techniques are conceived of as interpersonal methods aimed to stimulate, experience, understand and handle some specific subjective processes, then the traditional separation between diagnosis and therapy vanishes in many assessment procedures (Blatt 1975; Fischer 1994; Finn 2007). Projective techniques then can be seen as "empathy magnifiers" (Finn 2003, p. 126) and as initiators of a change process. In this sense we believe this study provides useful data for therapists who may adopt this therapeutic perspective to assessment and administer the ORT and other projective tests in their practice. It is useful for example to know that from the linguistic style of non-clinical subjects we can infer that chromatic colors trigger richer sensory information, and that more defined forms contribute to clear ideation and images. In order to foster the referential process of the patient, the therapist could choose such cards to elicit verbalizations that are more likely to evoke emotional experience of the central or problematic issues. Moreover, therapists can compare the IWRAD level of narratives told by a specific patient with the normative parameters of our non-clinical sample to detect significant deviations in specific type of cards.

From a strategic point of view the therapist could start by working collaboratively with the patient on narratives with high IWRAD levels, where images and narratives are accessible, and related to underlying issues with which he is more in contact. The projective measures might also reveal aspects of the patient’s experience that are more dissociated and less available; the therapist, and later the patient, could move toward accessing such issues when emotional structures have undergone some change.

### Limitations and Speculations

#### Variables Manipulation

A limitation of our study is the simultaneous manipulation of the color and form definition—as well as the interpersonal settings—in the 4 main cards groups (Blank card, Group A, B, and C). We cannot in fact distinguish the individual effect of each of them on the IWRAD measure. It is possible that the form and color components, if integrated, have an interactive rather than additive effect on the emotional engagement, as supported by some empirical results (Baughman 1967). Further experimental designs could better investigate the individual effects and interaction of these stimulus characteristics.

#### Human Silhouettes Effect

Examining the effect of the number of human silhouettes on the linguistic measures we found a noteworthy result that was not predicted. We see a decrease of the narrative IWRAD values from the 1-person silhouette to the cards with two silhouettes, with three silhouettes, with a group and the blank card. We may speculate that people can generate richer, more detailed and emotionally vivid narratives by imagining situations of themselves in relation to the single individual represented by the 1-person silhouette. On the other hand, it is possible that the viewer may grasp the signals coming from the multiple actors on a subsymbolic level and be emotionally involved in the situation but have more difficulty in connecting the experience to a narrative. The possibility that complex relational situations may evoke emotion but also activate processes of simplification, distancing or defense is supported in research by Ugazio et al. (2012). The effects of differences in number of figures also remains to be tested in future work.

#### The IWRAD_IWRRL Covariation

The results for the IWRAD_IWRRL covariation found in this study also raise a number of questions that require further consideration. Using the English version of the measure, this covariation is generally negative for patient language in therapy sessions, indicating the patient is involved *separately* in telling a story and reflecting on it (see Maskit 2021). In this study, the covariation was generally positive, and associated with well-defined stimulus features and chromatic colors, indicating a response to such cards of being immersed in a narrative and reflecting on it.

It is possible that this unexpected result might be a function of differences in language structure in Italian compared to English. However, application of the measure to Italian therapy sessions has shown the expected negative covariation. It is also possible that the positive covariation results from differences in the emotional and linguistic processes that are activated by projective techniques as compared to the inner processes of therapy. While patient speech consistently shows a negative covariation, substantial positive covariations have been found in responses to projective measures such as TAT cards using the English version (see Maskit 2021). These findings indicate that the Italian and English covariation measures are operating in a relatively similar way, and also raise interesting questions concerning the effects of different contexts on the playing out of the referential process. These questions will be examined on a clinical level in a subsequent paper.

This speculation concerning the effects of different stimulus contexts on the covariation is also supported by the finding of an average covariation close to zero (IWRAD_IWRRL = .0756) for the blank card. As discussed earlier, without perceptual stimuli, the individual must turn inward to generate a narrative. The experience of responding to the blank card may be seen as most similar to the experience of a therapy session in this respect.

## Conclusion

The results thus far have provided evidence for the ORT, combined with evaluation of the linguistic responses, as offering unique sources of information within the projective tests scenario. The breadth and systematic manipulation of the interpersonal settings prompted by the cards, combined with the effective use of colors and defined shapes, makes the ORT a powerful method offering “a uniquely structured assessment of what a person perceives, experiences, and feels, and the ways a person's relationships come into being" (Knafo 2010, p. 181).

This study shows systematic variability in IWRAD that is consistent with theoretical predictions and as such contributes to the convergent validity of the IWRAD measure. Future research should continue to explore how IWRAD and other measures of the referential process systematically vary in the responses to projective testing by clinical as well as non-clinical participants to better understand how different difficulties in mental life may be better, identified, addressed and understood.

## Footnotes

1 The manual for scoring the scales is also available in Italian: De Coro A., Caviglia G. (a cura di) (2000). *La*. *valutazione dell’Attività Referenziale*. Roma, Italy: Edizioni Kappa.
